# Comparative study on the pulmonary toxicity induced by some marketed pesticides after repeated oral administration in rats

**DOI:** 10.1038/s41598-026-62155-5

**Published:** 2026-07-19

**Authors:** Eman I. Hassanen, Marwa A. Ibrahim, Shaimaa Kamel

**Affiliations:** 1 Department of Pathology, Faculty of Veterinary Medicine, Cairo University, Giza, Egypt; 2 Department of Biochemistry and Molecular Biology, Faculty of Veterinary Medicine, Cairo University, P.O. Box 12211, Giza, Egypt

**Keywords:** Gene expression, Inflammation, Oxidative stress, Pesticides, Pulmonary toxicity, Biochemistry, Drug discovery

## Abstract

Mancozeb (MC), carbendazim (CBZ), and hymexazol (HML) are widely used fungicides in agricultural and veterinary fields worldwide. Our goal is to compare the pulmonary toxicity induced by oral gavage of these pesticides in rats. Thirty-five rats were used and divided into 7 groups as follows: (1) control negative, (2, 3) 125 and 250 mg/kg. bwt MC, respectively, (4, 5) 125, 250 mg/kg. bwt CBZ, respectively, (6, 7) 75, 150 mg/kg. bwt HML, respectively. All treatments were given to rats daily via oral routes for 14 days. All of these fungicides dose-dependently increased MDA levels and decreased GSH content in lung tissue homogenates. The oral intake of these fungicides could upregulate the mRNA levels of IL1β, TNFα, and NF-κB genes and exhibit strong iNOS and Cox-2 protein expressions along with severe histopathological alterations within the pulmonary tissues. The highest pulmonary toxicity was recorded in the HML groups, followed by the MC and CBZ groups. The oral intake of these fungicides dose-dependently induced pulmonary toxicity via oxidative stress, which is associated with triggering the NF-κB signaling pathway.

## Introduction

 Among the environmental pollutants, pesticide overexposure is one of the most important causes of pulmonary toxicity in humans and livestock^1^. The broad utilization of pesticides in agricultural and veterinary practices allowed high levels of these toxic chemicals to be present in the environment, causing a severe health risk to humans and animals^2^. Humans can be exposed to pesticides directly through dermal exposure or inhalation of the debris or indirectly through ingestion of residues present in food and drinking water^3^.

Pesticides like Mancozeb (MCZ), a manganese/zinc ethylene-bis-dithiocarbamate, are frequently used to guard field crops like vegetables, fruits, and grains from fungi^4^. Mancozeb has a variety of chronic adverse health effects that have been documented, despite its low acute toxicity (single dose of LD_50_) and limited environmental persistence^5^, including increased carcinogenic potential, disruption of the endocrine system, and toxic effects on the immune and neuronal systems^6,7^. Mancozeb has an oral LD_50_ greater than 4000 mg/kg bwt in rodents. It is rapidly absorbed via skin, lungs, and gastrointestinal tract and metabolized into ethylene urea that is distributed in various organs, leading to oxidative stress^8^. A recent study proved the ability of MCZ to initiate damage in the nasal mucosa of rats via activation of the inflammatory cytokine IL6^9^. Another chronic study affirmed moderate histopathological and ultrastructural changes in both trachea and lungs of nonmigratory pigeons living near an area sprayed with MCZ 8–12 times a year^10^.

Carbendazim (CBZ), a benzimidazole fungicide methyl N-(1 H-benzimidazol-2-yl) carbamate, is another widely used fungicide in agriculture^11,12^. Another use of CBZ is to preserve films, fibers, leather, fabric, elastic, polymers, and building materials^13^. Despite its toxicity, it is still widely used in agriculture in several nations, including Brazil and China^14^. According to reports, CBZ can increase the mutagenicity, carcinogenicity, and reproductive toxicity of germ cells^15^. Recent research has demonstrated that CBZ can cause rats to develop neurotoxicity^16,17^ and hepatocellular damage^18^. Additionally, a recent in vitro study revealed that CBZ induced alveolar epithelial damage through mitochondrial viability loss, caspase-3 activation, and free radical overgeneration^19^. A surveillance study in Eastern India discovered many respiratory symptoms in agricultural workers who sprayed carbamate pesticides, including runny nose, sore throat, dry cough, wheezing, chest pain, dyspnea, chronic bronchitis, and asthma that related to depletion of acetylcholinesterase (AchE)^20^. According to Ohayo-Mitoko et al., pesticides that block AchE, such as CBZ and MCZ, are linked to a higher prevalence of respiratory symptoms in Kenyan agricultural workers^21^. A study conducted by Ciesielski et al. on migrant farmworkers in North Carolina revealed a correlation between carbamate exposure and the presence of chest pain and respiratory distress^22^. Therefore, it is crucial to examine the detrimental effects of these pesticides on the lungs of experimental animals through all possible exposure pathways rather than direct inhalation.

Hymexazol (HML), an isoxazole fungicide (5-methyl-3(2 H)-isoxazolone), is also a broad-spectrum antifungal agent, soil disinfectant, and plant growth-regulating agent that is widely used in agriculture^23^. Hymexazol is highly efficacious and widely used in sugar beet, vegetables, rice, and other plants to control the seedlings and root rot disease^24,25^. However, its environmental toxicological assessment has not been well documented, and there is no report of its toxicity to rats and other experimental animals. We previously confirmed the neurotoxic and hepatorenal impact of HML in rats exposed to 1/40 LD50 HML^26,27^.

Concern over the dangers of these fungicides to the ecosystems and human health has persisted due to their presence of them in the environment and the possibility of exposure to them^28^. There are, however, few data on their toxicity to the lungs in experimental animals. Few studies have previously been conducted on the toxicity of ingested fungicides (MC, CBZ, and HML) in rats, and their findings have revealed that the reproductive organs, brain, endocrine gland, liver, and kidney are the target organs for their toxicity^29,30^. Consequently, the current study’s goal was to ascertain the impacts of oral dosing of sub-lethal levels of three broadly used fungicides (MC, CBZ, HML) on the pulmonary tissues of rats. In addition, we explored the possible mechanisms associated with these fungicides’ exposure from the molecular aspect and discussed the role of the NF-κB signalling pathway, iNOS, and COX.

## Materials and methods

### Chemicals

Carbendazim (50% Wettable Powder (WP)), Mancozeb (80% WP), and Hymexazol (30% Emulsion (ES)) were obtained from the Mammalian Toxicology Dept., Central Agricultural Pesticides Lab., Agriculture Research Center, Giza, Egypt. Based on the required concentration of active ingredients, both CBZ and MCZ were dissolved in sesame oil, while HML was dissolved in deionized distilled water.

### Animals and experimental design

Thirty-five adult male albino Wistar rats (170 ± 20 g) were obtained from the VACSERA in Helwan, Egypt. Animals were raised in plastic cages with access to unlimited amounts of water and regular commercial pelleted feed. Before use, they underwent a health checkup and two weeks of acclimatization to the research laboratory setting. The institutional animal care and use committee (IACUC) of Cairo University authorized all the experimental procedures (Approval No: Vet CU 2009 2022487), which were completed in compliance with the ARRIVE principles.

The following substances were given orally to rats every day for 14 days after being randomly assigned to one of seven groups (*n* = 5). Group 1 (control) was kept as a control negative group and given sesame oil as a baseline vehicle control. Groups 2 and 3 (MC1&MC2) were given MC at doses that corresponded to 1/40 and 1/20 of the LD_50_, respectively (125 and 250 mg active ingredient/kg bwt that equivalent to a human dose of 20- and 40 mg/kg bwt). Groups 4 and 5 (CBZ1&CBZ2) received CBZ at doses that corresponded to 1/40 and 1/20 of the LD_50_, respectively (125 and 250 mg active ingredient/kg bwt, that equivalent to a human dose of 20- and 40 mg/kg bwt). Groups 6 and 7 (HML1&HML2) received HML at doses that corresponded to 1/40 and 1/20 of the LD_50_, respectively (75 and 150 mg active ingredient/kg bwt, that equivalent to a human dose of 12- and 24 mg/kg bwt). There are two reasons for pesticide dose selection. The first reason is based on their LD_50_, which was reported to be 5000 mg/kg bwt for both MC and CBZ and 3000 mg/kg bwt for HML^31,32^. The second reason is based on several previous studies that assure the negative impact of these dosage levels on various organs of rats^33–35^. Moreover, male Wistar rats were selected for this study to eliminate the confounding effects of cyclical hormonal fluctuations associated with the female estrous cycle, thereby ensuring a homogenous experimental group for optimal baseline toxicological comparison. Sesame oil was chosen as the control vehicle due to its minimal metabolic impact, unlike water, and to adhere to the 3R principles for animal welfare by reducing animal usage. Distilled water is inert substance with no physiological or toxicological lung effects. So, comparing hymexazol to the sesame oil group accurately assessed hymexazol’s toxicity.

### Sampling

Rats were euthanized after 14 days by cervical dislocation following anaesthesia to obtain lung tissue samples that were split into two portions. The first portion was kept at −80 °C until it was employed for gene and oxidative stress analysis, whereas the second portion was fixed in 10% neutral buffered formalin for histology and immunohistochemistry.

### Assessment of the lung oxidant/antioxidant status

According to Beutler et al.^36^ and Ohkawa et al.^37^, the supernatant of lung tissue homogenate was employed for the measurement of GSH and MDA, respectively. The reduced glutathione was assessed using the commercial kit of Spectrum. The absorbance was measured at 405 nm. Whereas the levels of malondialdehyde (MDA) were assessed by mixing the supernatant of lung homogenate (200 µL) with thiobarbituric acid (TBA) (1 mL). The tubes were kept in a boiling water bath for 30 min, cooled, and read at 535 nm.

### Histopathological examination

To obtain paraffin-embedded tissue sections, formalin-fixed lung tissue specimens were drained using escalating grades of ethanol, cleansed with xylene, impregnated in paraffin wax, and sectioned at 4.5 μm. These sections were then stained with H&E and examined under a light Olympus microscope BX43 to look for any pathological changes and captured by an Olympus DP27 camera linked to CellSens Dimensions software (Product Version, 1.13; Core Version, XV 3.12 (Build 13479)) https://www.olympus-lifescience.com/en/software/cellsens/^38^.

According to the procedure outlined by Passmore et al.^39^, all the observable pathological parameters were assessed using an ordinal semiquantitative scoring system. Histopathological scoring was performed on at least 5 microscopic fields per section, across 5 distinct sections per group, representing 5 individual rats per group (*n* = 5). The scores from the multiple microscopic fields for each animal were averaged to yield a single data point per rat, meaning the individual animal remained the true statistical unit in all downstream analyses. The criteria of bronchial and bronchiolar epithelial degeneration and necrosis, luminal inflammation, interstitial and alveolar edema, hemorrhage, inflammation, perivascular inflammation and vascular congestion were graded as follows: (0) normal histology, (1) mild < 10% tissue damage, (2) moderate 11–25% tissue damage, (3) severe 26–50% tissue damage, and (4) extensive severe > 50% tissue damage. Furthermore, the focal lesions, such as multifocal inflammatory cell aggregation, perivascular cuffing, and vascular congestion, were graded as follows: (0) no foci/field; (1) < 3 foci/field; (2) 3–6 foci/field; (3) 7–12 foci/field; (4) > 12 foci/field^40^.

### Immunohistochemical staining

Avidin-biotin-peroxidase complex (ABC) was used in an immunohistochemical analysis to identify iNOS and Cox-2 as markers for inflammation in lung tissue sections. Briefly, different primary antibodies (Abcam Ltd., USA) were incubated with deparaffinized tissue sections before the reagents needed for the ABC reaction were incubated with them (Vectastain ABC-HRP Kit, Vector Laboratories). After that, slides were marked with peroxidase and colored with DAB-chromogen substrate (Sigma), and then examined under a light Olympus microscope. Image J software was used to calculate the mean percentage area of different immunostaining reactions in various experimental groups. At least 3 microscopic fields per animal section were quantified, across 5 distinct sections per group, representing 5 individual rats per group (*n* = 5). The field-level measurements for each individual rat were averaged to yield a single data point per rat, meaning the individual animal remained the true statistical unit in all downstream analyses.

### Estimation of the transcript level of the target genes Il1β, Tnf-α and Nf-κB

 The total RNA was extracted from the lung tissue using the RNeasy mini kit (Qiagen). We assessed the purity and concentration of each sample using a NanoDrop. Afterwards, the first-strand cDNA was synthesized^41^ using the RevertAid First Strand cDNA Synthesis (Thermo Scientific #K1622). The quantitative real-time PCR was performed using a Real-Time PCR System (Applied Biosystems, USA). The thermal cycler was programmed for 40 cycles of denaturation at 95 °C for 30 s, annealing at 58 °C, and extension at 72 °C for 30s. The ACTB gene was used as the internal standard to normalize the expression values^42^. The primer sets used in the study are shown in Table 1. Each sample was performed in triplicate. Furthermore, no-template controls were used. The CT values were used to calculate the gene/ACTB ratio, with a value of 1.0 used as the control (calibrator). The normalized expression change was calculated by 2^−∆∆CT^^43^.

**Table 1:** The primer sets for the target genes.

Gene	Sense	Antisense	Amplicon size	Melting temperature	Accession no
Nf-κB	ACCTGGAGCAAGCCATTAGC	AGTTCCGGTTTACTCGGCAG	234	60	NM_199267.2 https://www.ncbi.nlm.nih.gov/nuccore/NM_199267.2
Tnf-A	ACACACGAGACGCTGAAGTA	GGAACAGTCTGGGAAGCTCT	235	59	NM_012675.3 https://www.ncbi.nlm.nih.gov/nuccore/NM_012675.3/
Il-1β	TTGAGTCTGCACAGTTCCCC	GTCCTGGGGAAGGCATTAGG	161	59.8	NM_031512.2 https://www.ncbi.nlm.nih.gov/nuccore/NM_031512.2
Actb	CCGCGAGTACAACCTTCTTG	CAGTTGGTGACAATGCCGTG	297	59	NM_031144.3 https://www.ncbi.nlm.nih.gov/nuccore/NM_031144.3

NF-κB, nuclear factor kappa-light-chain-enhancer of activated B cells; TNF-α, tumor necrosis factor alpha; IL-1β, Interleukin-1 beta; ACTB, beta actin housekeeping genes.

### Statistical analysis

 The means and the standard error of means (SEM) were used to represent all of the parametric data. The statistical package programme (SPSS version 25) was used to analyze the recorded results using one-way analysis of variance (ANOVA) and Duncan’s post hoc test; P values of 0.05 or less are considered statistically significant. The histopathological scoring’s nonparametric values were reported as medians and examined using the Kruskal-Wallis H test and the Mann-Whitney U test.

## Results

### Assessment of the lung oxidant/antioxidant status

**Fig. 1:**
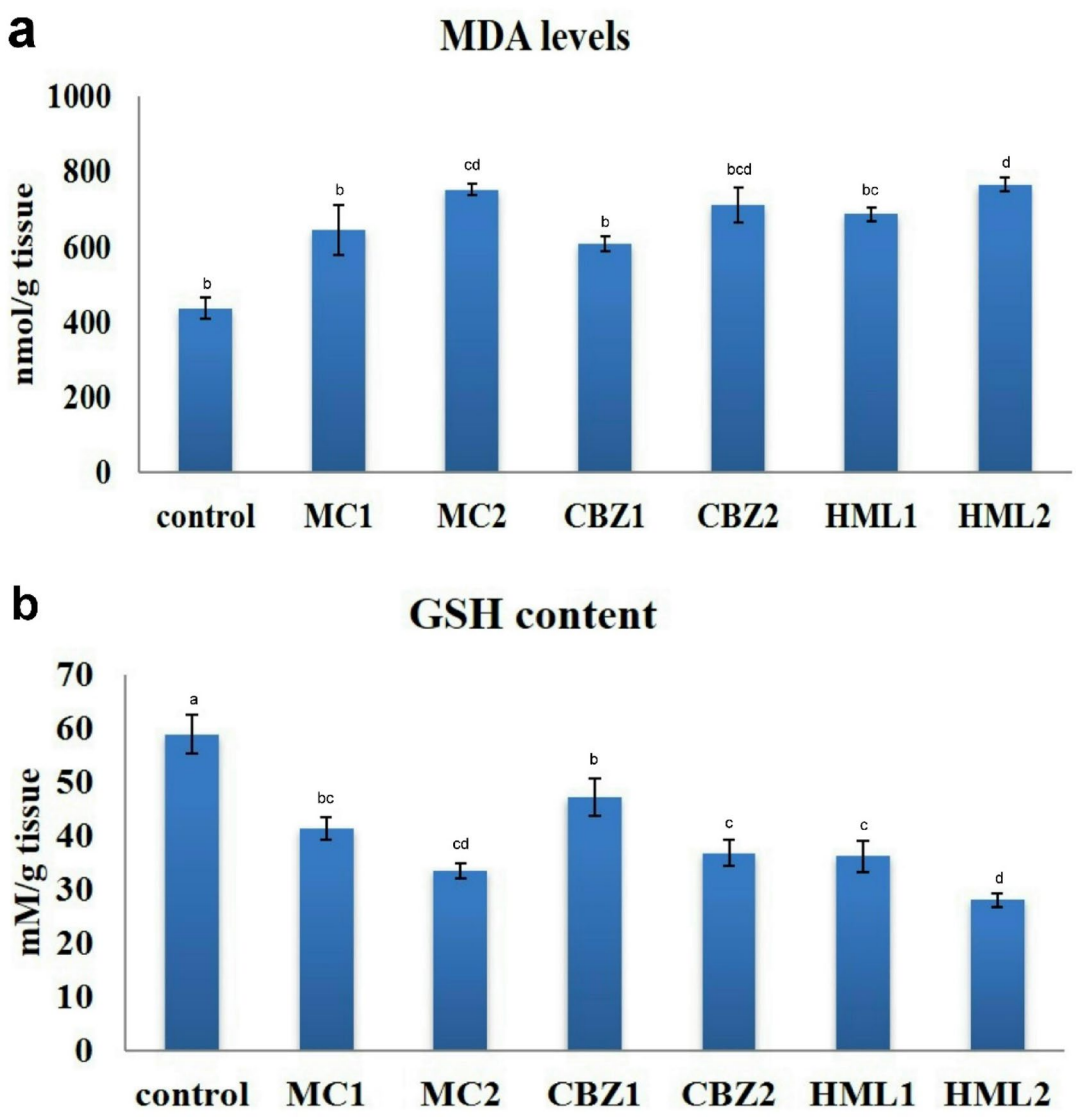
Bar chart representing the effect of daily oral administration of the studied pesticides on the levels of MDA (a) and GSH (b) in lung tissues of rats. Data are expressed as Mean ± SEM. (*n* = 5), values with different superscript letters (**a–d**) differ significantly at *P* ≤ 0.05.

The MDA levels displayed significant elevations in all the pesticide-treated groups, whereas the high doses of MC and HML recorded higher levels than the low doses (Fig. 1a). Administration of both doses of MC, CBZ, and HML significantly reduced the GSH content among all treated groups, such that the lesser contents were shown in the groups that were exposed to the high doses of the three pesticides (Fig. 1b).

**Fig. 2:**
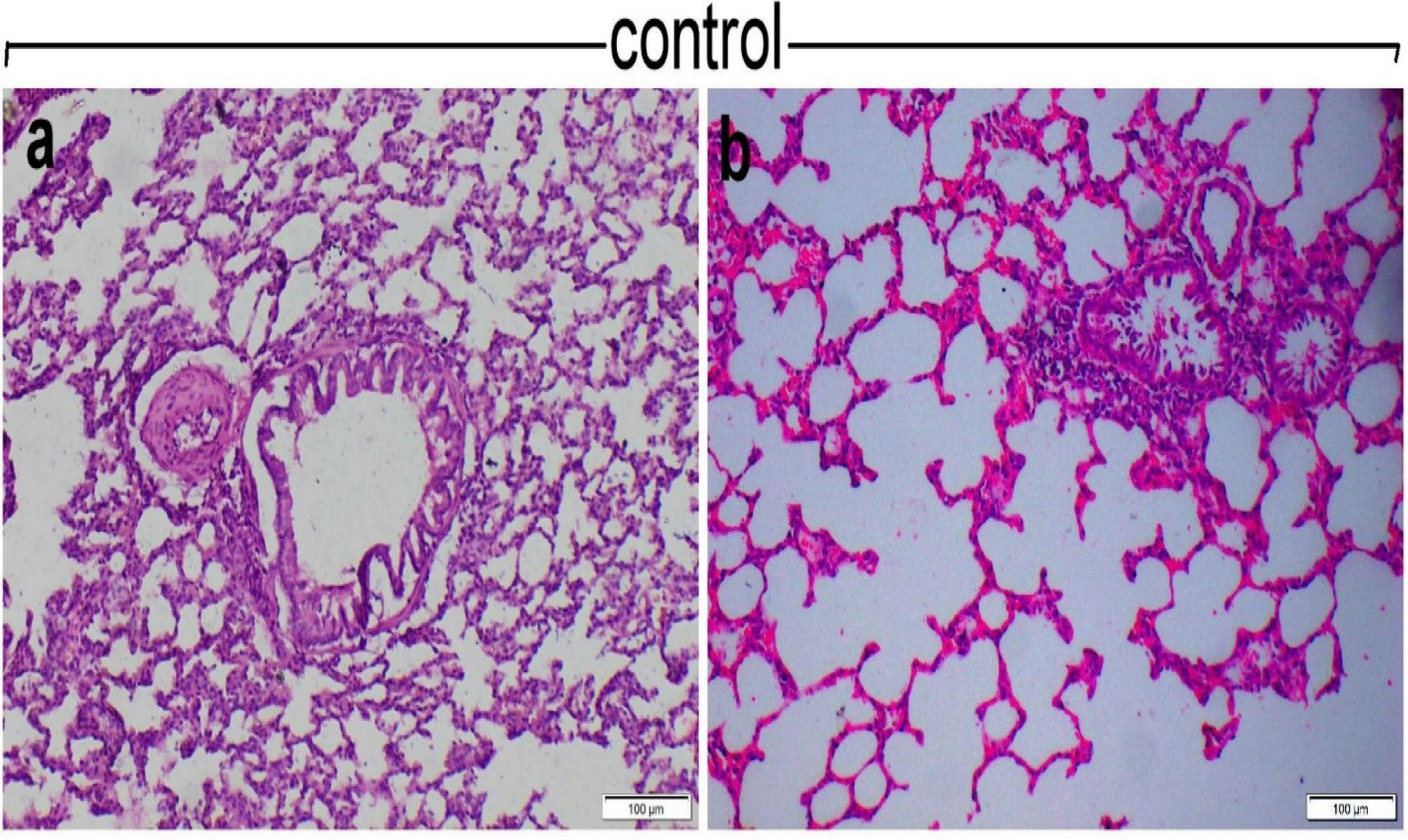
Photomicrograph of lung sections stained with H&E representative of the negative control group. (**a,b**) Control rats showing normal histological architecture.

**Fig. 3:**
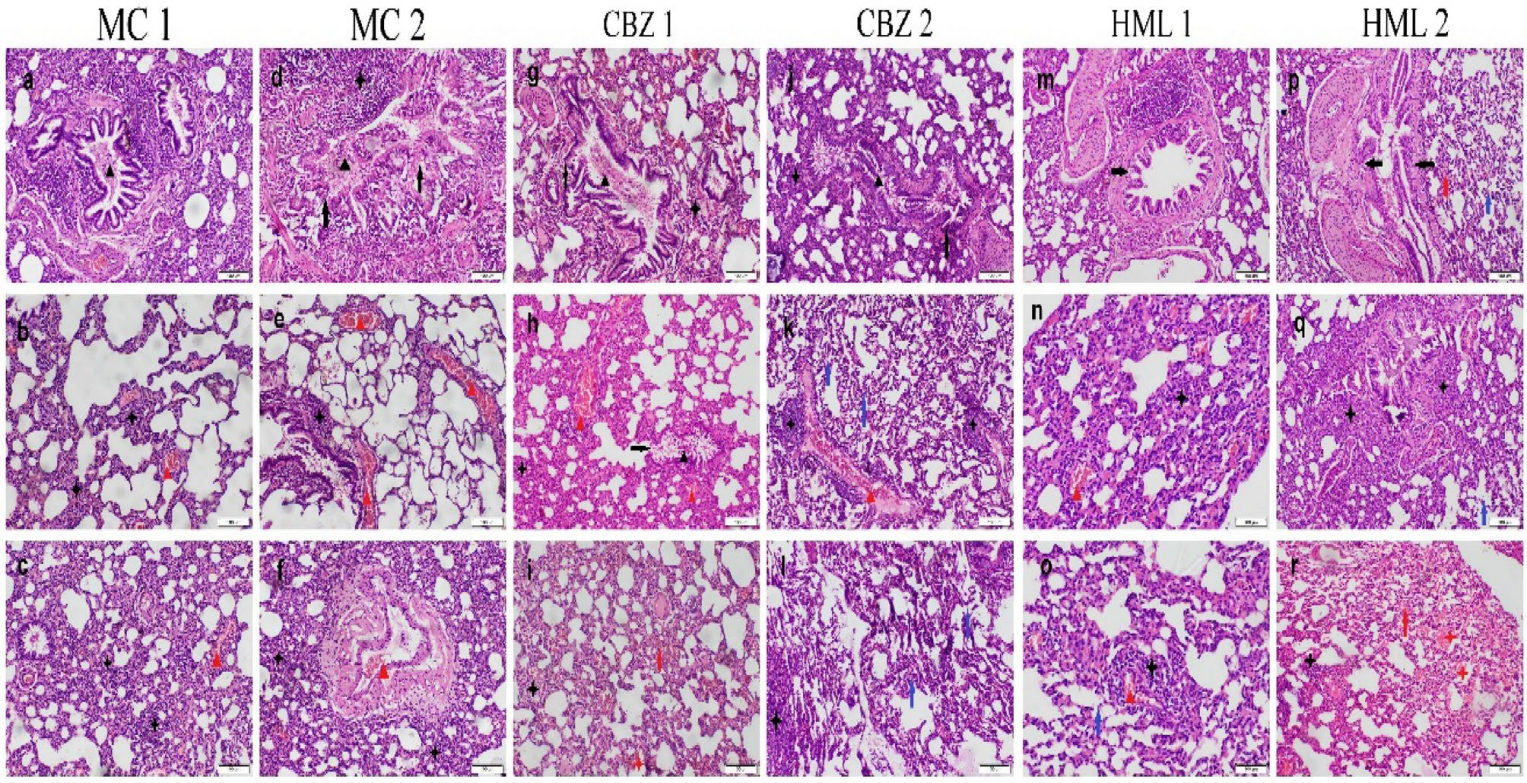
Photomicrograph of H&E stained lung tissue sections representing; (**a–c**) group receiving 125 mg MC (MC 1), (**d–f**) group receiving 250 mg MC (MC 2), (**g–i**) group receiving 125 mg CBZ (CBZ 1), (**j–l**) group receiving 250 mg CBZ (CBZ 2), (**m–o**) group receiving 75 mg HML (HML 1), (**p–r**) group receiving 150 mg HML (HML 2). Note: (black arrows) necrosis and degeneration of the epithelial lining the bronchi/bronchioles, (black triangles) desquamation of the lining epithelium mixed with inflammatory cells, (black stars) interstitial inflammatory cells infiltration, (red triangles) vasculitis, (red arrows) interstitial hemorrhage, (red stars) alveolar hemorrhage, (blue arrows) alveolar damage.

### Histopathological examination

Lung tissue sections obtained from the control group showed normal histological organization of pulmonary bronchi, bronchioles, and alveoli (Fig. 2). There were dose-dependent histopathological alterations in the lung tissues obtained from all pesticide-treated groups. The group receiving 125 mg MC showed moderate histopathological alterations. There was an inflammatory exudate in the bronchial lumen (Fig. 3a). Prominent thickening of the alveolar septa by inflammatory cell infiltration was the most prominent lesion observed (Fig. 3b). The microscopic lesions become more severe in group receiving 250 mg MC and there were extensive degeneration and necrosis in the epithelial lining the bronchi and bronchioles (Fig. 3 d). Some sections showed extensive alveolar damage and some alveoli were filled with inflammatory cells and necrotic cell debris (Fig. 3e). The blood vessels showed vasculitis associated with perivascular edema and inflammatory cells infiltration (Fig. 3f). Concerning CBZ receiving group, there were mild histopathological lesions manifested by few inflammatory cell infiltrations in the bronchial lumen (Fig. 3 g) and moderate interstitial inflammation with moderate thickening of the alveolar septa (Fig. 3h). On the other hand, the group receiving 250 mg CBZ showed severe pathological changes. Most bronchi and bronchioles showed necrosis of their epithelial lining with severe inflammatory cell infiltration in their lumen (Fig. 3j). There was extensive alveolar damage along with marked vascular and perivascular inflammation (Fig. 3k). Remarkable pathological changes were also observed in the HML receiving group, in a dose-dependent manner. Most bronchi/bronchioles showed goblet cell hyperplasia/metaplasia associated with luminal inflammatory exudates (Fig. 3 m,p-q). Interstitial tissues showed severe congestion and extensive inflammatory cell aggregations (Fig. 3n,q). Some alveoli collapsed while others overdistended with air, forming giant alveoli along with extensive alveolar hemorrhage (Fig. 3r).

The results of the microscopic lesion scoring were summarized in Table 2 and revealed the highest score in all parameters in the group receiving a high dose of HML, followed by those receiving the high dose of either MC or CBZ. The scores of low-dose pesticide-receiving groups were quite similar to each other but showed a significant increase compared to the control group.

**Table 2:** The microscopic lesion scoring for pulmonary structures in all groups.

	Control	MC1	MC2	CBZ1	CBZ2	HML1	HML2
Vascular lesions							
Congestion	0^a^	2^c^	3^d^	1^b^	4^e^	2^c^	4^e^
Perivascular inflammation	0^a^ 0^a^	0^a^	3^c^	1^b^	4^d^	2^c^	4^d^
Vasculitis		1^b^	1^b^	2^c^	4^d^	1^b^	4^d^
Interstitial lesions							
Edema	0^a^	1^b^	1^b^	0^a^	2^c^	2^c^	2^c^
Hemorrhage	0^a^	2^b^	3^c^	0^a^	4^d^	2^b^	4^d^
Inflammatory cells	0^a^	2^b^	4^c^	2^b^	4^c^	2^b^	4^c^
Bronchial and bronchiolar lesions							
Epithelial degeneration and necrosis	0^a^	1^b^	2^c^	3^d^	4^e^	2^c^	4^e^
Luminal inflammation	0^a^	3^c^	4^d^	3^c^	4^d^	2^b^	4^d^
Alveolar lesions							
Edema	0^a^	1^b^	1^b^	0^a^	2^c^	1^b^	2^c^
Hemorrhage	0^a^	1^b^	2^c^	0^a^	3^d^	2^c^	4^e^
Destruction	0^a^	1^b^	3^d^	1^b^	2^c^	2^c^	3^d^
Luminal inflammation	0^a^	1^b^	3^d^	1^b^	2^c^	2^c^	3^d^

Data are expressed as Median. (*n* = 5 rats/group). Values with different superscript letters (a-e) differ significantly at *P* ≤ 0.05.

### Immunohistochemical staining

**Fig. 4:**
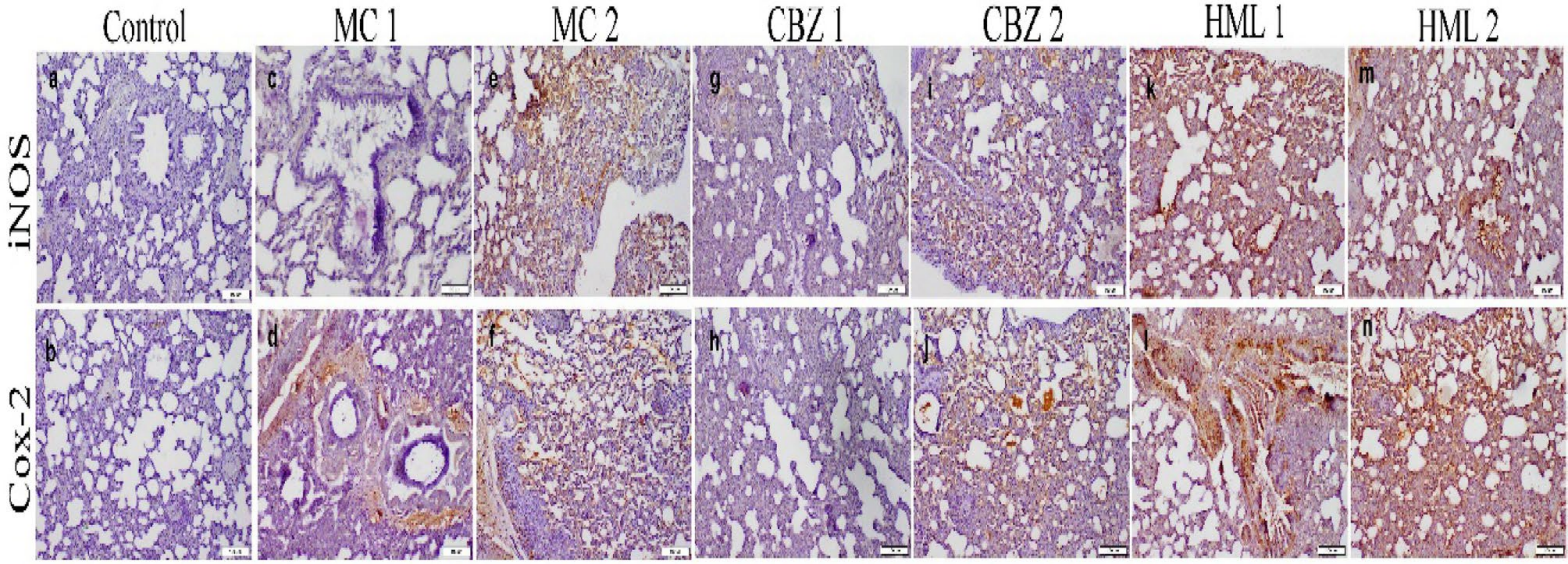
Photomicrograph representing the immunostaining expression of iNOS and Cox-2 in lung tissue sections of various experimental groups. (**a,b**) control group with negative immunostaining expression of both markers. (**c,d**) group receiving 125 mg MC (MC 1) showed negative iNOS expression with moderate positive Cox-2 expression. (**e,f**) group receiving 250 mg MC (MC 2) showed strong iNOS and Cox-2 expression. (**g,h**) group receiving 125 mg CBZ (CBZ 1) showed mild immunopositivity of both immune markers. (**i,j**) group receiving 250 mg CBZ (CBZ 2) showed strong immunopositivity of both immune markers. (**k–l**) group receiving 75 mg HML (HML 1) and (**m,n**) group receiving 150 mg HML (HML 2) showed strong immunopositivity of both markers.

The control group displayed negative iNOS and Cox-2 expression in lung tissues, while all pesticide-receiving groups showed variable degrees of iNOS and Cox-2 expression in lung tissue sections. Strong immunopositivity of the above-mentioned immune markers was recorded in the group receiving 250 mg MC and those receiving 150 mg HML. Localization of both immune markers was mainly in the alveolar pneumocyte type 1, followed by the interstitial inflammatory cells and epithelial cells lining the bronchial mucosa. Moreover, other MC and HML receiving groups showed moderate immunopositivity of the previously mentioned immune markers. CBZ-receiving groups at doses of 125 or 250 mg showed mild to moderate protein expression of both immune markers (Fig. 4).

The results (summarized in Table 3) recorded a significant increase in both iNOS and Cox-2 immunopositivity percentage area in all pesticide-receiving groups, comparable to the control group. The highest mean % area of both immune markers’ expression was recorded in all over the pulmonary tissues of the group receiving either 250 mg MC or 150 mg HML, compared with other groups. Other pesticide-receiving groups showed values nearly similar to each other.The results (summarized in Table 3) recorded a significant increase in both iNOS and Cox-2 immunopositivity percentage area in all pesticide-receiving groups, comparable to the control group. The highest mean % area of both immune markers’ expression was recorded in all over the pulmonary tissues of the group receiving either 250 mg MC or 150 mg HML, compared with other groups. Other pesticide-receiving groups showed values nearly similar to each other.

**Table 3:** The mean % area of the examined immune markers in lung tissue.

	Control	MC1	MC2	CBZ1	CBZ2	HML1	HML2
iNOS mean percentage area (%/microscopic field)							
BEC	0.0	0.0	2.5 ± 0.7^b^	0.5 ± 0.2^a^	2.8 ± 1.4^b^	10.5 ± 3.5^c^	12.1 ± 2.5^c^
AP-2	0.0	1.5 ± 0.3^a^	15.1 ± 2.3^c^	5.2 ± 0.5^b^	13.5 ± 3.5^c^	25.9 ± 4.9^d^	30.3 ± 3.7^e^
IIC	0.3 ± 0.1^a^	2.2 ± 0.7^b^	2.1 ± 0.5^b^	2.2 ± 0.9^b^	5.7 ± 1.2^c^	15.1 ± 2.3^d^	20.5 ± 2.8^e^
Cox-2 mean percentage area (%/microscopic field)							
BEC	0.0	0.0	8.1 ± 1.5^b^	0.0	4.3 ± 0.2^a^	5.1 ± 0.8^a^	9.4 ± 2.2^c^
AP-2	0.0	5.6 ± 0.6^a^	20.1 ± 2.7^c^	4.5 ± 0.5^a^	15.2 ± 2.2^b^	20.1 ± 5.5^c^	25.4 ± 4.8^d^
IIC	0.5 ± 0.1^a^	2.6 ± 0.5^b^	9.5 ± 1.1^d^	1.5 ± 0.1^b^	5.5 ± 0.3^c^	10.1 ± 2.7^d^	10.4 ± 2.9^d^

Data are expressed as Mean ± SEM. (*n* = 5 rats/group). Values with different superscript letters (a-d) differ significantly at *P* ≤ 0.05.

### Estimation of the transcript level of the target genes IL1β, TNF,α, and NF-κB

**Fig. 5:**
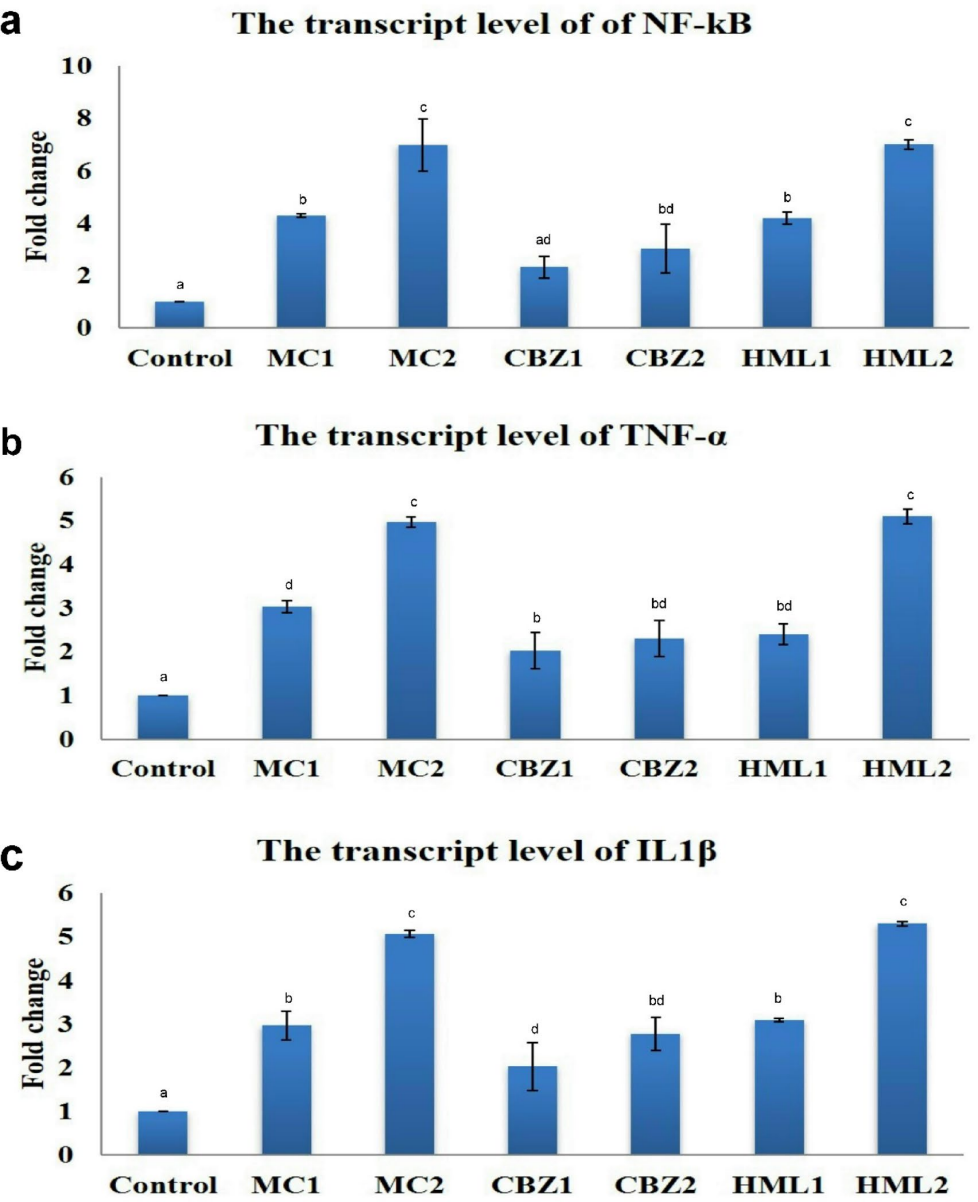
Bar chart representing the effect of daily oral administration of the studied pesticides on the m-RNA levels of NF-κB (a), TNF-α (b), and (c) IL-1β in lung tissues of rats. Data are expressed as Mean ± SEM. (*n* = 5), values with different superscript letters (**a–d**) differ significantly at *P* ≤ 0.05.

The results of the mRNA levels of the target genes were shown in Fig. 5, demonstrating that the administration of MC, CBZ, and HML upregulated the transcription of NF-κB, TNF,α, and IL-1β significantly compared to the control group. Whereas, the administration of the high dose of MC and HML showed more potent effects on the expression levels of the target genes compared to the groups given their low doses, and those who received both doses of CBZ.

## Discussion

Pesticides such as mancozeb (MC), carbendazim (CBZ), and hymexazol (HML) have been used in agriculture to kill plant fungi and control disease vectors^44^. The frequent usage of these fungicides pollutes the environment, prompting widespread public concern^34^. Furthermore, residues of these fungicides have been found in soil, feed, and food items^45,46^. As a result, the human body is exposed to these fungicides in three ways: dermal and oral exposure in all humans, or inhalation by agricultural workers, especially in countries where pesticide safety procedures are not in place^47,48^. The previous studies confirmed the harmful impact of the studied fungicides on some organs of rats following oral intake, including the brain, liver, kidneys, and reproductive organs, but little data are available about their pulmonary intoxication. Therefore, the current study’s goal is to elucidate the adverse effects of repeated ingestion of these fungicides on the pulmonary tissue of rats.

In the present study, oral exposure to the tested fungicides resulted in a dose-dependent manner in malondialdehyde (MDA) levels accompanied by a significant depletion of reduced glutathione (GSH) in pulmonary tissue homogenates, indicating the induction of oxidative stress. Because the alveoli are surrounded by a network of capillaries, these fungicides and their metabolites may have penetrated the lung tissues via systemic circulation. Furthermore, the presence of pulmonary metabolizing enzymes, including cytochrome P450 mixed-function oxidases and flavin-containing monooxygenases, suggests that these compounds may undergo biotransformation within the lung, potentially contributing to their toxic effects^49^. Both enzymes use NADPH and oxygen to produce highly reactive metabolites that are linked to oxidative tissue damage^50^. Furthermore, numerous pesticides interfere with mitochondrial activity and adenosine triphosphate (ATP) generation by targeting mitochondrial enzymes^51^. In humans and animals, oxidative stress plays a key role in pesticide-induced toxicity^52^. Pesticides increase the production of reactive oxygen species (ROS) by disrupting the oxidant/antioxidant equilibrium, which is linked to mitochondrial dysfunction^53^. The overproduction of ROS, such as superoxide or hydrogen peroxide, promotes lipid peroxidation, protein breakdown, and nuclear damage, all of which contribute to cell death^54,55^. Reduced glutathione (GSH) is an intracellular antioxidant that glutathione peroxidase uses to detoxify ROS^56^. In the current investigation, all administered fungicides resulted in a significant drop in lung GSH content, indicating a cellular reactivity to ROS in response to oxidative stress. Surprisingly, the HML group had the lowest GSH content and the highest MDA levels, indicating that HML has a higher oxidative defense impairment than the other fungicides. Our histological findings of alveolar and bronchial epithelial degeneration and necrosis may be supported by these findings.

All of the fungicides investigated were linked to pulmonary inflammation, either directly via pro-inflammatory cytokines or indirectly through the creation of ROS^57^. Inflammation was manifested by strong immunopositivity of iNOS and Cox-2 protein expression along with upregulation of NF-κB, IL-1β, and TNF-α. These findings were in line with the histopathological findings, which revealed perivascular and interstitial mixed inflammatory cell infiltration along with vasodilation. We observed that the macrophages were the predominant inflammatory cells in most sections. There is a positive correlation between the processes of oxidative stress and inflammation. During inflammation, ROS produced by the activated macrophages is thought to play a role in further cell membrane damage^58^. Furthermore, ROS is associated with inflammation as a correlation to the release of cytokines, including IL-1β, TNFα, and IF-γ, which promote the attraction of more neutrophils and macrophages. As a result, free radicals are important correlated mediators in the initiation and maintenance of the inflammatory process^59,60^. The promotion of pulmonary inflammation, macrophage activation, and acute lung injury is potentially linked to the upregulation of pro-inflammatory cytokines (IL-1β, TNF-α) and overexpression of iNOS and Cox-2 immune markers^61–63^. Because activated macrophages express elevated cytokines, macrophages are thought to play a key role in pesticide-induced inflammation in rats. These findings are supported by the in vitro data acquired from macrophage cell lines exposed to carbamate pesticides^64^. The activated macrophages can also be linked to the expression of iNOS and Cox-2, resulting in excessive production of nitric oxide (NO) and prostaglandins (PG), respectively, which is potentially linked to further cell membrane damage and inflammation^65^. The membrane lipid peroxidation leads to the release of arachidonic acid (AA) metabolites. Cox-2 converted the AA metabolites to thromboxane, prostacyclin, and prostaglandins in various cell types, causing vasodilation and increased vascular permeability^66^. Despite its role as an oxygenase, Cox-2 creates superoxide, which causes oxidative stress, free radical-induced cell damage, and inflammation^67^. Regarding iNOS, it is an inducible enzyme that is produced by various cells in response to cytokines (IL-1β, TNF-α)^68^. It is present in various cells, including macrophages, endothelium, smooth muscle cells, and respiratory epithelium. This enzyme can synthesize the free radical gas (NO) that causes vasodilatation and binds with other free radicals, forming more toxic NO derivatives and leading to extensive cell damage^69^. On the other side, previous studies reported that the carbamate pesticides (CBZ, MC) inhibit the acetylcholine esterase (AchE) via binding with it, forming carbamyl-AchE, causing the accumulation of acetylcholine within the lung tissue, resulting in bronchial asthma, mucous oversecretion, vasodilatation, and promoting inflammatory reactions^70,71^. This data may support our histopathological findings of vasodilation, interstitial inflammatory cell infiltrations, hyperplasia of goblet cells with excessive mucous secretion, degeneration, and necrosis of some bronchial and bronchiolar epithelium.

NF-κB is a well-known protein transcription factor that regulates the process of inflammation^72^. The activation of the NF-κB signal transduction pathway is required for the expression of the cytokines described above^73,74^. In fact, when cytokine receptors, pattern recognition receptors, or T- and B-cell receptors are activated, the IκB kinase (IKK) complex is stimulated, resulting in IκBα phosphorylation and degradation. Once in the nucleus, the NF-κB dimers p50/RelA and p50/c-Rel then act as transcription factors to activate target genes at the κB response element^75^. The nuclear translocation of NF-κB leads to upregulation of several genes involved in the process of inflammation, such as Cox-2 and IL^76^. According to a previous study, carbendazim activates the NF-κB signal transduction pathway in the liver, kidney, and brain of rats, which is in line with our findings^77^. In this study, we provided interesting results regarding the influence of Mancozeb, carbendazim, and hymexazol on lung tissue. Based on the aforementioned data, the repeated oral intake of these three pesticides demonstrated pulmonary toxicity in a dose-dependent manner. All of them induced oxidative stress and upregulated the mRNA levels of IL1β, TNFα, and NF-κB genes and exhibited strong iNOS and Cox-2 protein expressions.

The study has many limitations, which should be considered in future investigations. First, our design focused on a short-term, high-dose oral gavage model to characterize sub-acute hazard profiles. This model does not reflect real-world environmental exposure scenarios. Further studies on the effect of long-term, low doses should be conducted. Second, while we observed correlations between pesticide exposure and oxidative/inflammatory markers (MDA, GSH, IL-1β, TNF-α, NF-κB, iNOS, and Cox-2), these findings are descriptive, not causal, indicating a need for future research into the underlying mechanisms. Finally, this study evaluated pulmonary toxicity exclusively in male rats to minimize hormonal variability. Since pesticide metabolism and inflammatory sensitivity can exhibit sex-dependent differences, future studies incorporating female cohorts are warranted to comprehensively evaluate the gender-specific risks of these fungicides. Broader transcriptome-level profiling would enable the identification of additional dysregulated signaling pathways and potential mechanistic targets. Also, it is highlighted as an important future direction for further elucidating the underlying mechanisms. We suggest that future studies incorporating NF-κB nuclear translocation or pathway inhibitors are required to confirm a direct causal link between oxidative stress and activation of the NF-κB pathway.

## Data Availability

The datasets generated during and/or analysed during the current study are available from the corresponding author on reasonable request.

## Abbreviations

BEC: Bronchial epithelial cells
AP-2: Alveolar pneumocyte type-2
IIC: Interstitial inflammatory cells
